# Cactus in Functional Affections of the Heart

**Published:** 1891-12-26

**Authors:** 


### THE PRACTITIONER'S MIRROR AND RETROSPECT.

[*The Editor will be glad to receive offers of co-operation and contributions from members of the profession. All letters should be addressed to* The Editor, The Lodge, Porchester Square, London, W.]

### MEDICINE.

### CACTUS IN FUNCTIONAL AFFECTIONS OF THE HEART.

We have in a previous issue referred to this new remedy and indicated some of its theraputical uses.

Of the order cactaceæ, the plant is described by Mr. F. G. Sultan, Ph.D., as having a branching, fleshy, green, five or six angled stem. The angles are beset with clusters of five or six short radiating spines. The numerous imbricated calyx lobes are linear, acute, brownish, the inner ones yellow. The flowers, which open in the night, are very fragrant and produce an orange-coloured, internally white berry, the size of an egg. Mr. Sultan has succeeded in obtaining the active principle of the plant, which he calls cactina, the flower of this species being the part richest in this active principle, yielding over two per cent.

The physiological action of cactina has been recently investigated by Dr. Myers of Rochester.* When administered to dogs in toxic doses, a primary rise in arterial pressure and pulse rate is observed, followed by a secondary diminution in both. The cardiac pulsations become arhythmical, and the heart finally becomes arrested in systole. After section of the vagi the arterial pressure and pulse rate were both increased by an injection of two centigrammes of the drug and a strong faradic current applied to the cut end of the vagus failed to arrest the heart's action. But even after both the vagi and sympathetic nerves were divided an intravenous injection of cactina maintained a decided rise in arterial pressure, from which Myers fairly concludes that the effects on the pulse are the result of direct stimulation of the intra-cardiac accelerator ganglion. But that the rise in blood pressure was also partly due to stimulation of the medullary vaso-motor centres was proved by the sudden diminution of the increase in blood pressure in a dog under the influence of cactina on section of the spinal cord high up. It is also found to have a generally stimulating action on the spinal motor nerves. Cactina applied to the conjunctiva is absolutely unirritating, but the crude drug applied to the skin is capable of producing excoriations and the formation of pustules. These physiological investigations explain clinical experience with cactus. Dr. Watson Williams has pointed out † that it is especially in functional disorders of the heart that cactus will be found useful, in these cases he now rarely prescribes any other cardiac remedy. In the distressing palpitation from reflex irritation in dyspepsia it hardly ever fails to relieve. One of his most remarkable cases was that of a highly neurotic lady about forty-five years of age, to whom he was hastily summoned in the middle of the night "as she was dying." Arriving at the house he found her last will and testament being rapidly prepared for her signature. The patient in bed, unable to lie down, complaining of a terrible beating of the heart, and a sense of impending of death. She had a rapid but forcible pulse, and the heart sounds were perfectly clear. He was able to reassure the patient, and promise a speedy recovery. She was quickly relieved by small doses of cactus. Pitzer‡ claims that in sexual exhaustion nothing gives such speedy relief as cactus, for it strengthens the cardiac plexus of the sympathetic, and improves the cardiac nutrition. The action of cactus on the motor nerve centres of the spine, as well as on the heart, explains its satisfactory employment in such cases. In fact, all these cases of palpitation and rapid heart action being relieved by a drug like cactus, which Myers found increased the rapidity of the pulse, are explained when we remember that they are the result of exhaustion, and, therefore, hyper-excitability of the accelerator nerves of the heart. A small dose of cactus has a tonic action on the exhausted cardiac enervation, and thus the rapid and often intermittent or irregular pulse becomes lessened in frequency. But an abnormally slow and feeble pulse may be quickened and strengthened by the same drug stimulating the cardiac ganglia and muscle, and if it be pushed, may give rise to a very rapid pulse, the same drug thus having apparently opposite effects in different conditions. Myers considers that a special indication for its use is during the critical periods of adynamic fevers, as it combines the elements of a heart and spinal motor stimulant; in this he confirms the experience of Engstad.* In the "tobacco heart," the palpitation of hearts hypertrophied from prolonged and excessive exercise are excellent cases for this remedy. Aulde, of Philadelphia says, "we are often confronted with patients who suffer from exhaustion, with irregular and intermittent pulse, due to the abuse of tobacco, and here, instead of unpalatable remedies like nux vomica, we have cactus, which is rather agreeable than otherwise to most persons, and the effect upon the system and the circulatory apparatus is quickly apparent. The patient should take five to ten drops three times daily. The same treatment is indicated for the relief of a similar condition which often affects those accustomed to the inordinate use of tea. The active principle, theine, it has been found, will cause the heart to intermit, but in cactus we have a drug which meets this emergency."

* *New York. Med. Journ.*, June 13, 1891.

† *Practitioner*, October 1891.

‡ *Amer. Med. Journ.*, 1884.

